# Health Impacts of Natural Background Radiation in High Air Pollution Area of Thailand

**DOI:** 10.3390/toxics12060428

**Published:** 2024-06-13

**Authors:** Narongchai Autsavapromporn, Chutima Kranrod, Rawiwan Kritsananuwat, Phachirarat Sola, Pitchayaponne Klunklin, Imjai Chitapanarux, Churdsak Jaikang, Tawachai Monum, Masahiro Hosoda, Shinji Tokonami

**Affiliations:** 1 Division of Radiation Oncology, Department of Radiology, Faculty of Medicine, Chiang Mai University, Chiang Mai 50200, Thailand; pitchayaponne.kl@cmu.ac.th (P.K.); imjai.chitapanarux@cmu.ac.th (I.C.); 2 Institute of Radiation Emergency Medicine, Hirosaki University, Hirosaki 036-8564, Japan; kranrodc@hirosaki-u.ac.jp (C.K.); m_hosoda@hirosaki-u.ac.jp (M.H.); tokonami@hirosaki-u.ac.jp (S.T.); 3 Natural Radiation Survey and Analysis Research Unit, Department of Nuclear Engineering, Faculty of Engineering, Chulalongkorn University, Bangkok 10330, Thailand; rawiwan.kr@chula.ac.th; 4 Thailand Institute of Nuclear Technology, Nakhon Nayok 26120, Thailand; phachirarats@tint.or.th; 5 Toxicology Section, Department of Forensic Medicine, Faculty of Medicine, Chiang Mai University, Chiang Mai 50200, Thailand; churdsak.j@cmu.ac.th (C.J.); tawachai.m@cmu.ac.th (T.M.); 6 Graduate School of Health Science, Hirosaki University, Hirosaki 036-8564, Japan

**Keywords:** lung cancer, natural background radiation, natural radionuclides, radon, thoron

## Abstract

Chiang Mai province of Thailand is known for having the highest natural background radiation in the country, as well as being recognized as one of the world’s most polluted cities for air quality. This represents the major contributor to the development of lung cancer. This research aims to estimate the comprehensive dose of both internal and external exposure due to natural background radiation and related health perspectives in the highly polluted area of Chiang Mai. The average values of indoor radon and thoron concentrations in 99 houses over 6 months were 40.8 ± 22.6 and 17.8 ± 16.3 Bq/m^3^, respectively. These results exceed the worldwide value for indoor radon and thoron (40 and 10 Bq/m^3^), respectively. During burning season, the average values of indoor radon (56.7 ± 20 Bq/m^3^) and thoron (20.8 ± 20.4 Bq/m^3^) concentrations were higher than the world-wide averages. The radon concentration in drinking water (56 samples) varied from 0.1 to 91.9 Bq/L, with an average value of 9.1 ± 22.8 Bq/L. Most of the drinking water samples (87%) fell below the recommended maximum contamination limit of 11.1 Bq/L. The average values of natural radionuclide (^226^Ra, ^232^Th and ^40^K) in 48 soil samples were 47 ± 20.9, 77.9 ± 29.7 and 700.1 ± 233 Bq/kg, respectively. All values were higher than the worldwide average of 35, 30 and 400 Bq/kg, respectively. The average value of outdoor absorbed gamma dose rate (98 ± 32.5 nGy/h) exceeded the worldwide average of 59 nGy/h. Meanwhile, the average activity concentrations of ^226^Ra, ^232^Th and ^40^K in 25 plant food samples were 2.7 ± 0.1, 3.2 ± 1.6 and 1000.7 ± 1.9 Bq/kg, respectively. The ^40^K concentration was the most predominant in plant foods. The highest concentrations of ^226^Ra, ^232^Th and ^40^K were found in Chinese cabbage, celery and cilantro, respectively. The total annual effective dose for residents in the study area varied from 0.6 to 4.3 mSv, with an average value of 1.4 mSv. This indicates a significant long-term public health hazard due to natural background radiation and suggests a heightened radiation risk for the residents. The excess lifetime cancer risk value (5.4) associated with natural background radiation was found to be higher than the recommended value. Moreover, the number of lung cancer cases per year per million average of 25.2 per million persons per year was in the limit range 170–230 per million people. Overall, our results will be used for future decision making in the prevention of lung cancer risk associated with natural background radiation.

## 1. Introduction

Air pollution such as ambient particulate matter (PM) is one of the major environmental problems in northern Thailand. Chiang Mai, the largest city in this region, experiences elevated levels of PM during the dry burning season (from December to April), leading to its designation as one of the world’s most polluted areas. PM in Chiang Mai originated from the burning of agricultural biomass or forest fires and traffic emission [1]. Prolonged exposure to PM has been associated with various health diseases such as respiratory diseases, cardiovascular diseases, and lung cancer (LC) [2]. LC has been the major cause of cancer deaths in Chiang Mai province in the past few decades, as reported by the World Health Organization (WHO) [3,4]. Although tobacco smoking is a significant risk factor for LC among Chiang Mai residents, other risk factors such as natural background radiation, e.g., uranium (^238^U, with half-life 4.47 × 10^9^ y), thorium (^232^Th, with half-life 1.4 × 10^10^ y) potassium (^40^K, with half-life 1.23 × 10^9^ y) and air pollution (such as carcinogenic polycyclic aromatic hydrocarbons) also contribute to LC [2,5,6,7].

Radon (^222^Rn) and its progeny is the second leading cause of LC after tobacco smoking and is the primary cause of LC in nonsmokers. Radon is considered a human carcinogenic agent (Group I) and the main source of natural background radiation. It is radioactive gas generated from radium (^226^Ra) produced in the decay chain of ^238^U with a long half-life of 3.82 days and it emits alpha particles. It comes from natural gas, water, soil and building materials [5,6,7,8]. Additionally, thoron (^220^Rn) generated by alpha particles from the ^232^Th decay series with a short half-life of 55.6 s also contributes to the annual effective dose for the general population [9]. When combined with PM in the air, these gases can be deposited in the human respiratory tract via alpha particle emission, leading to an increased risk of the development of LC within 5 to 25 years [8]. According to the WHO, there is a 16% increase in risk of LC per 100 Becquerels (Bq)/m^3^ in measured indoor radon concentration in homes with prolonged exposure [10]. Generally, the WHO recommends an action level of indoor radon concentration of 100 Bq/m^3^ [5]. Our previous findings on indoor radon concentration in Chiang Mai were higher than the nation average of 16 Bq/m^3^ [5,8]. Notably, indoor radon concentrations in Chiang Mai during burning season (high levels of PM) were higher than those in non-burning season [11,12]. Future studies are required to confirm these findings.

Radon can dissolve and accumulate in water from underground sources. Thus, the ingestion of high radon concentrations in drinking water through the gastrointestinal system and inhalation of radon from water sources serve as primary pathways for radon entering the human body [13,14]. The United States Environmental Protection Agency (USEPA) estimated that 89% of lung cancer death caused by breathing radon in indoor air from water and 11% of stomach cancer deaths caused by the ingestion of radon in drinking water [14]. The USEPA recommended radon concentration in drinking water should not be above the maximum contamination level (MCL) of 11.1 Bq/L [14]. Prolonged exposure to low or high radon concentrations in drinking water can contribute to stomach cancer, emphasizing the need to assess radon levels in drinking water.

Naturally occurring radioactive material (NORM) is the radioactive elements found in the environment, including humans, air, water, soil and food. NORM that mostly comes from the decay chain of ^238^U and ^232^Th and their decay products, such as ^40^K [15,16,17]. These radionuclides are usually transferred to the environment and transmitted into the human body through the ingestion pathway via contaminated food, leading to long-term internal radiation exposure. Terrestrial radionuclides present in soil are transferred to various plants (e.g., vegetables, herbs and fruits). Plants obtain these radionuclides from soil through root uptake and deposit them in leaves and seeds, which are then consumed by humans, thus entering the food chain [18]. Hence, it is important to study the radioactive contamination of food plants and to estimate ^226^Ra (^238^U)-, ^232^Th- and ^40^K-specific activities in the soil to evaluate the health risk of the general population. ^226^Ra is a daughter product of the ^238^U decay series and is the most important; it is used to determine the overall radioactivity for the ^238^U decay series. According to the United Nations Scientific Committee on the Effects of Atomic Radiation (UNSCEAR), worldwide average activity concentrations of ^238^U, ^232^Th and ^40^K nuclides are 35, 30 and 400 Bq/kg [19]. Chiang Mai is a highland region that is rich in natural resources including rice, vegetables, maize, medical plants, herbs and fruits. Recently, it was found that soils in Chiang Mai have a higher activity concentration of ^226^Ra, ^232^Th and ^40^K than the national and worldwide averages [20]. However, there are limited data available regarding natural radioactivity (^226^Ra, ^232^Th and ^40^K), particularly in the high-air-pollution area of Chiang Mai.

Kong Khaek is a subdistrict of Mae Chaem district located in the west part of Chiang Mai Province. It is surrounded by forested landscapes and mountains including Doi Inthanon, Thailand’s highest peak. Maize (corn) is the major crop grown in Kong Khaek. Consequently, the burning of maize crop residue and forest is a major cause of air pollution in Chiang Mai. Recently, Mae Chaem has been identified as one of the areas in Chiang Mai with the highest concentration of ambient particulate matter that are 2.5 μm or less in diameter (PM_2.5_) due to many hot spots of haze pollution from burning agricultural biomass and forest. Therefore, this paper is the first study on natural background radiation across the entirety of the Kong Khaek subdistrict. We conducted measurements to access both external and internal radiation dose to the residents due to natural radioactive sources. The primary objective of this study is to investigate indoor radon and thoron concentrations within residential houses, the consumption of radon concentration in drinking water and the natural radioactivity content from the soil and plants. Furthermore, we aim to estimate the total annual inhalation dose, total annual ingestion dose, total annual effective dose, the excess lifetime cancer risk (ELCR) and risk of LC due to exposure to natural radioactive sources for residents within this study area.

## 2. Materials and Methods

### 2.1. Study Area

**Figure 1:**
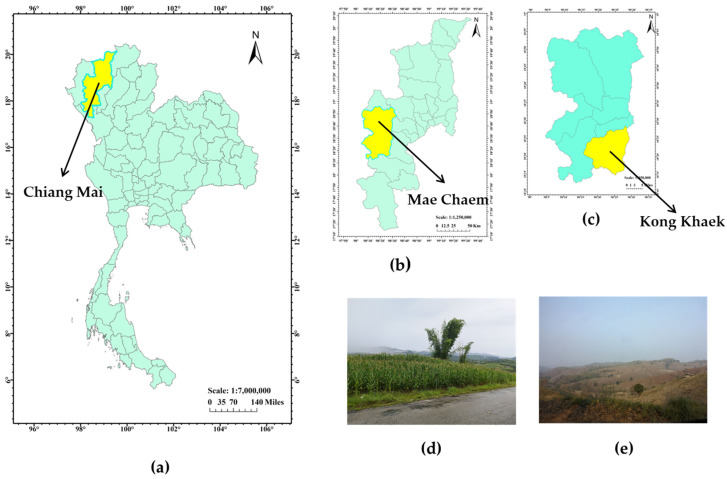
Map of the study area. (**a**) Map of Chiang Mai in upper northern Thailand. (**b**) Map of Mae Cheam district. (**c**) Map of Kong Khaek subdistrict. (**d**) Maize (corn) plantation and (**e**) bald mountain with maize cropping in Kong Khaek subdistrict.

The study area was Kong Khaek, which is a subdistrict of Mae Chaem district in Chiang Mai province located in the upper northern of Thailand (Figure 1). The area is a plateau in the granite highland mountains in the Mae Cheam forest reserve. Forests cover approximately 80% of the total area. The subdistrict contains 12 villages with a population of 6566 people in 2018. The geographical coordinates of this study are 18°16′38″ and 18°26′48″ N and within 98°21′36″ and 98°27′23″ E, covering an area of 288 km^2^. Kong Khaek has three seasons, which are a winter season from November to February, summer from March to May, and rainy season from June to October. Kong Khaek subdistrict produces a large amount of maize, one of the biggest producers in Mae Cheam district. Consequently, burning the maize waste causes hazardous haze pollution during the dry season in the study area.

### 2.2. Long-Term Indoor Radon and Thoron Concentrations Measurement

**Figure 2:**
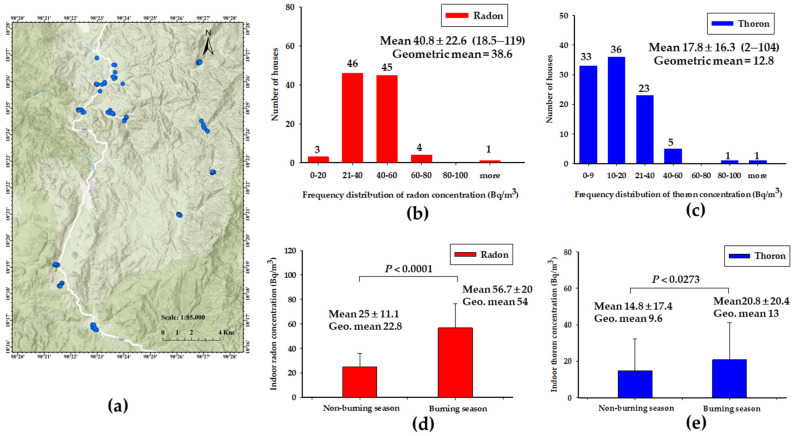
Indoor radon and thoron concentrations in Kong Khaek subdistrict. (**a**) Study area and RADUET detector locations. Frequency distribution of indoor radon (**b**) and thoron (**c**) concentration in 12 villages of Kong Khaek subdistrict. Variation of indoor radon (**d**) and thoron (**e**) concentrations during non-burning and burning seasons.

A passive type of radon–thoron discriminative monitor using a solid-state track detector (CR-39) called a RADUET (Radosys Ltd., Budapest, Hungary) was used to determine the indoor radon and thoron concentrations throughout Kong Khaek subdistrict (12 villages) from September 2022 to March 2023 (Figure 2a). A total of 99 randomly selected houses (total of 198 RADUET detectors) were analyzed for a period of 6 months, in which the detectors were replaced at 3-month intervals to cover the non-burning season (September–November, PM_2.5_~15.4 µg/m^3^) and burning season (December–March, PM_2.5_~61.7 µg/m^3^). The RADUET detectors were set in the center of the bedroom in each house, at least 150 cm above the floor and 50 cm away from the door and window. Most of the selected houses were built of wood with a concrete floor and good ventilation condition. After exposure, a CR-39 chip was chemically etched for 6 h in 6.25 M NaOH solution at 90 °C, and alpha tracks were counted with an automatic reading system.

The indoor radon (C_Rn_) and thoron (C_Tn_) concentrations were calculated using Equations (1) and (2).

(1) $$C_{\mathrm{RN}}=\frac{\rho }{\mathrm{kt}}$$

(2) $$C_{\mathrm{TN}}=\frac{\rho }{\mathrm{kt}}$$

where ρ is an alpha track density (track/cm^2^) corrected by the background track density, k is the conversion factor from alpha track density to radon or thoron concentrations (track/cm^2^/h)/(Bq/m^3^) and t is the exposure time (h) [11].

The annual effective dose received by the residents in the study area from inhalation of radon (H_Rn_) and thoron (H_Tn_) concentrations were estimated using Equations (3) and (4) based on the UNSCEAR report [19].

H_Rn_ (mSv) = C_Rn_ × F_Rn_ × O × T × D_Rn_ (3)

H_Tn_ (mSv) = C_Tn_ × F_Tn_ × O × T × D_Tn_ (4)

where C_Rn_ and C_Tn_ are the average radon and thorn concentrations (Bq/m^3^), respectively.

F_Rn_ and F_Tn_ are equilibrium factors for radon and thoron and their progeny, respectively, and F_Rn_ and F_Tn_ values are 0.4 and 0.02, respectively. O is the occupancy factor (0.8), T is an average exposure period (8760 h), D_Rn_ is the dose coefficient for radon (9 × 10^−6^ mSv/h per Bq/m^3^) and D_Tn_ is an inhalation dose coefficient for thoron (40 × 10^−6^ mSv/h per Bq/m^3^).

### 2.3. Radon Concentration in Drinking Water

**Figure 3:**
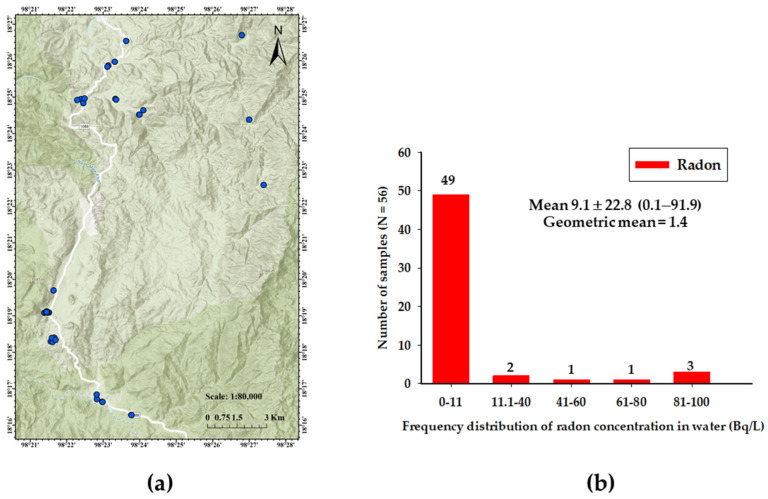
Radon concentration in drinking water. (**a**) Location of the sampling sites. (**b**) Frequency distribution of radon concentration levels in drinking water.

We collected drinking water from 56 sample points from different areas of Kong Khaek subdistrict within the time interval of September 2022 to March 2023 (Figure 3a). Two glass bottles (250 mL) of water sample were collected for each sample point excluding any air bubbling. The concentration of radon in the collected drinking samples was measured using the RAD-H_2_O accessory of the RAD7, an electrostatic collection type radon monitor, manufactured by Durridge Company, Billerica, MA, USA. The RAD7 was thoroughly dried out before analyzing with a larger desiccant to absorb the moisture (humidity less than 6%). We used the WAT250 protocol in 5 min cycles in 5 recycle time, and an identical procedure was repeated 5 min later to measure alpha activity. This process was completed 30 min after water sample collection. Thus, a correction-measured concentration of radon from the sample collection was applied [21]. Generally, the radon activity concentration is calculated based on the equation of radioactive decay, as in Equation (5).

A = A_O_ *e*^−(λt)^ (5)

where A_O_ is the initial activity concentration of radon in Bq/L, A is the measured activity concentration of radon in Bq/L, λ is the radon decay constant per minute and t is the period between sample collection and measurement.

The annual effective dose received by the residents in the study area from ingestion of drinking water (H_ingw_) was estimated according to the International Commission on Radiological Protection (ICRP), UNSCEAR and WHO report, using Equation (6) [22,23].

H_ingw_ (μSv) = C_Rn_ × Cw × D_ing_ (6)

where C_Rn_ is the average radon concentration in drinking water measured by RAD7 (Bq/L), C_w_ is the annual water consumption in L (~2 L/day, 730 L) and DCF_ing_ is the dose coefficient of ingested radon for adults (3.5 × 10^−6^ mSv/Bq).

The annual effective dose received by the residents in the study area from inhalation of radon degassing from water (H_Rnw_) was calculated using Equation (7).

H_Rnw_ (μSv) = C_Rn_ × R_aw_ × O × T × D_Rn_ (7)

where C_Rn_ is the average radon activity concentration in drinking water measured by RAD7 (Bq/L), R_aw_ is transfer factor of radon from water (10^−4^) [24], O is the occupancy factor (0.8), T is an average exposure period (8760 h), and D_Rn_ is the dose coefficient for radon (9 × 10^−6^ mSv/h per Bq/m^3^).

### 2.4. Natural Radionuclide Activity Concentrations in Soil

**Figure 4:**
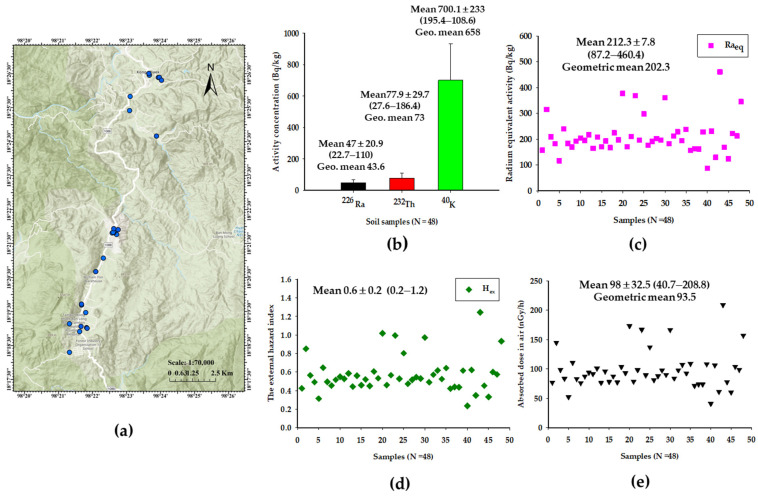
Natural radionuclide activity concentrations in soil samples. (**a**) Soil sampling location in the study area. (**b**) Activity concentration of natural radionuclides in soils. (**c**) Comparison of radium equivalent activity in the soil samples. (**d**) Comparison of the external hazard index in the soil samples. (**e**) Comparison of absorbed dose rate in outdoor air in the soil samples.

A total of 48 soil samples (about 0–20 cm depth of surface soil) were collected randomly from different areas of Kong Khaek subdistrict from September 2022 to March 2023 (Figure 4a). Collected soil samples of 1 kg were packed in plastic bags. Subsequently, samples were dried in an oven at 110 °C until constant weight (>24 h), pulverized to a fine powder and sieved through a 250 μm mesh. Each sample was then weighed and packed into airtight plastic containers. All samples were allowed to rest undisturbed for one month, allowing the decay products in the soil to reach a radioactive secular equilibrium.

After one month, the activity concentrations of natural radionuclide (^226^Ra, ^232^Th and ^40^K) were determined using one of two high-purity germanium (HPGe) detectors with relative efficiencies of 25 and 30% in a low background configuration. Energy and efficiency calibrations of the HPGe detector were measured using three different International Atomic Energy Agency (IAEA) standard reference materials (IAEA-RGU-1, IAEA-RGTh-1 and IAEA-RGK-1). Counting time for soil sample was set at 86,400 s. The full energy-absorbed peaks of ^214^Bi (609.3 keV) and ^228^Ac (911.2 keV) were used for the calculation of ^226^Ra and ^232^Th activity concentrations, respectively. The single peak of 1460.8 keV was used directly for the calculation of ^40^K activity concentration [20].

Radium equivalent activity (Ra_eq_) is the radiation hazard index associated with the natural radionuclide. It is a weighted sum of activities concentration of ^226^Ra, ^232^Th and ^40^K based on the 370 Bq/kg of ^226^Ra, 259 Bq/kg of ^232^Th and 4810 Bq/kg of ^40^K, which produced the same gamma dose rate [19]. It is calculated by Equation (8).

Ra_eq_ (Bq/kg) = C_Rn_ + 1.43 C_Th_ + 0.077C_k_ (8)

where C_Rn_, C_Th_ and C_k_ are the concentrations of ^226^Ra, ^232^Th and ^40^K in Bq/kg, respectively.

The external hazard index (H_ex_) was used to evaluate the harmful effect of gamma radiation from natural radioactive nuclide. The H_ex_, Equation (9), is as below [19],

H_ex_ = (C_Rn_/370) + (C_Th_/259) + (C_k_/4810) (9)

where C_Rn_, C_Th_ and C_k_ are the concentrations of ^226^Ra, ^232^Th and ^40^K in Bq/kg, respectively.

The external exposure of public from terrestrial radionuclides in the soil can be characterized by absorbed dose rate in air (D_a_). Gamma absorbed dose rate in outdoor air at 1 m above ground surface can be determined by Equation (10) [19].

D_a_(nGy/h) = 0.462C_Ra_ + 0.604C_Th_ + 0.0417C_k_ (10)

where C_Ra_, C_Th_ and C_k_ are the concentrations of ^226^Ra, ^232^Th and ^40^K in Bq/kg, respectively.

The outdoor annual effective dose (H_O_) was derived from the absorbed dose rate in air by the residents in the study area using a conversion factor of 0.7 Sv/Gy, which is used to convert to absorbed rate, H_O_, with an outdoor occupancy of 0.2. It was calculated with Equation (11) [19,25].

H_O_ (mSv) = D_a_ × T × O × D_O_ (11)

where D_a_ is the absorbed dose rate in air (nGy/h), T is the average exposure period (8760 h), O is the occupancy factor (0.2) and D_O_ is the dose conversion factor (0.7 Sv/Gy).

### 2.5. Natural Radionuclide Activity Concentrations in Plant Foods

**Figure 5:**
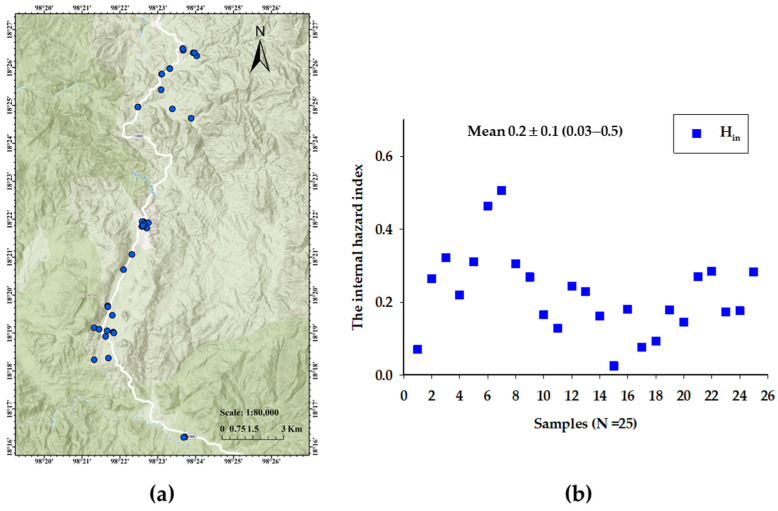
Natural radionuclide activity concentrations in plant samples. (**a**) Map of sampling lo-cations. (**b**) Comparison of the internal hazard index in plant foods.

A total of 52 samples from 25 types of plant (vegetables, fruits and herbs) were collected randomly from different areas of Kong Khaek subdistrict including in the local market from September 2022 to March 2023 (Figure 5a, Table 1). All samples were washed with clean water three times to remove soil and dust. Samples were then dried in the oven at 70–80 °C for 6 h. All samples were ground into a fine powder and sealed in airtight plastic containers to reach a radioactive secular equilibrium for at least one month. After one month, all samples were analyzed for natural radionuclide activity concentration (^226^Ra, ^232^Th and ^40^K) with a HPGe detector using the same method to measure natural radionuclide activity concentrations in soil. It should be noted that radionuclide activity concentration (^226^Ra, ^232^Th and ^40^K) for all plant samples was measured for 48 h.

The internal hazard index (H_in_) was used to evaluate the harmful effect of internal exposure to natural radioactive nuclide. To calculate, the H_in_, Equation (12) was used [19].

H_in_ = (C_Rn_/185) + (C_Th_/259) + (C_k_/4810) ≤ 1 (12)

where C_Rn_, C_Th_ and C_k_ are the concentrations of ^226^Ra, ^232^Th and ^40^K in Bq/kg, respectively.

The annual effective dose received by the residents in the study area due to the consumption of food (H_ingf_) was calculated using Equation (13) [19].

H_ingf_ (mSv) = C_p_ × A_i_ × D_ingf_ (13)

where C_p_ is the annual amount of food consumed (kg/y) based on consumption data of Thailand, where daily consumption of vegetables and fruits was 276 g (~100 kg/y) [26], A_i_ is the activity concentration of each natural radionuclide (Bg/kg) and D_ingf_ is the dose coefficient for ingestion of food (^226^Ra; 2.8 × 10^−4^ mSv/Bq, ^232^Th; 2.3 × 10^−4^ mSv/Bq and ^40^K; 6.2 × 10^−7^ mSv/Bq) [19].

### 2.6. Health Risk Assessment

#### 2.6.1. Total Annual Effective Dose (H)

H is the total annual effective dose due to internal and external exposures of residents in the study area. The H can be estimated using Equation (14) as below [19].

H (mSv) = H_Rn_ + H_Tn_ +H_Rnw_+ H_ingw_ + H_O_ + H_ingf_ (14)

where H_Rn_ is the annual effective dose from inhalation of radon, H_Tn_ is the annual effective dose from inhalation of thoron, H_Rnw_ is the annual effective dose from the inhalation of radon degassing from water, H_ingw_ is the annual effective dose from the ingestion of drinking water, H_O_ is the outdoor annual effective dose due to external gamma radiation exposure (H_O_) and H_ingf_ is the annual effective dose due to the ingestion of food.

#### 2.6.2. The Annual Equivalent Dose to Lung (H_L_)

Equation (15) was used to calculate the H_L_ [27].

H_L_ (mSv) = (H_inh_ + H_ing_) × W_R_ × W_T_ (15)

where (H_inh_ + H_ing_) is total annual equivalent dose from inhalation and ingestion (H_Rn_ + H_Tn_ + H_w_ + H_O_ + H_ingw_ + H_ingf_), W_R_ (radiation-weighting factor) is 20 for alpha particles and WT (tissue weighing factor for lung) is 0.12.

#### 2.6.3. The Annual Equivalent Dose to Stomach (H_s_)

H_s_ was calculated using Equation (16) as below [27].

Hs (mSv) = H_ing_ × W_R_ × W_T_ (16)

where H_ing_ is the total annual equivalent dose due to ingestion (H_ingw_ + H_ingf_), W_R_ (radiation-weighting factor) is 20 for alpha particles and WT (tissue weighing factor for stomach) is 0.12.

#### 2.6.4. Excess Lifetime Cancer Risk (ELCR)

ELCR is the estimation of cancer risk for residents in the study area due to natural background radiation. Equation (17) was used [28].

ELCR = H × DL × RF(17)

where H is total annual effective dose, DL is the average lifespan for Thai people (77 years) and RF is the fatal risk factor (0.05 Sv^−1^).

#### 2.6.5. The Number of Lung Cancer Cases per Year per Million (LCC)

LCC was estimated using Equation (18) as below [29,30].

LCC = H × RFLC(18)

where H is total annual effective dose and RFLC is the risk of LC induction per million per person (18 × 10^−6^ mSv^−1^ y).

### 2.7. Statistical Analysis

Statistical analysis was performed using Sigma Plot 10 software (Sigma, St. Louis, MO, USA). All data presented were analyzed based on arithmetic mean (average) ± standard derivation. Student’s *t* test and Wilcoxon signed rank test were used to estimate the difference between the two groups. A *p* value of 0.05 or less was considered statistically significant. We used ArcGIS Pro software version 3.0.1 (ESRI, Redlands, CA, USA) to create maps.

## 3. Results

### 3.1. Long-Term Indoor Radon and Thoron Concentration Measurements

Figure 2b,c represent the frequency distribution of indoor radon and thoron concentrations in 99 houses within the Kong Khaek subdistrict during the period September 2022 to March 2023, respectively. The estimated values of indoor radon concentration in the bedroom varied from 18.5 to 119 Bq/m^3^ with an average value of 40.8 ± 22.6 Bq/m^3^, while indoor thoron concentrations ranged from 2 to 104 Bq/m^3^, with an average value of 17.8 ± 16.3 Bq/m^3^. About 50% and 97% of the houses in the study area had indoor radon concentrations higher than the worldwide average of 40 Bq/m^3^ and national average of 16 Bq/m^3^, respectively (Figure 2b) [5]. Notably, only one of the surveyed houses had an indoor radon concentration higher than the permissible level of 100 Bq/m^3^ as recommended by the WHO. Moreover, around 67% of houses in this study area had indoor thoron concentrations higher than the worldwide average of 10 Bq/m^3^ (Figure 2c) [5]. These findings indicate that the Kong Khaek subdistrict has higher radon and thoron concentrations compared to the global average value.

To further investigate the association between indoor radon/thoron concentrations and high air pollution. We needed to obtain indoor radon and thoron concentrations in Kong Khaek subdistrict during non-burning (September–November 2022) and burning (December 2022–March 2023) seasons. Figure 2d,c show a significant statistical difference (*p* < 0.0001 and 0.0273) between indoor radon and thoron concentrations during non-burning and burning seasons, respectively. The average values of indoor radon and thoron concentrations were higher during the burning period (56.7 ± 20 and 20.8 ± 20.4 Bq/m^3^) than in the non-burning period (25 ± 11.1 and 14.8 ± 17.4 Bq/m^3^), respectively. This study demonstrates that indoor radon and thoron concentrations during the burning period exceeded the worldwide average values in areas with high pollution.

### 3.2. Radon Concentration in Drinking Water

A study was conducted to assess the level of radon concentrations in drinking water collected from the Kong Khaek subdistrict between September 2022 and March 2023. The frequency distribution of radon concentration in 56 drinking water samples is shown in Figure 3b. The radon concentration in water varied from 0.1 to 91.9 Bq/L, with an average of 9.1 ± 22.8 Bq/L. The average values of radon concentration in water (almost 87%) were within the maximum contaminant level by the USEPA (11.1 Bq/L). However, the study found that 13% of water samples exceeded the concentration limit of 11.1 Bq/L as recommended by the USEPA but remained below the reference level of 100 Bq/L as reported by WHO [14,23].

### 3.3. Natural Radionuclide Activity Concentration in Soil

The activity concentrations of ^226^Ra, ^232^Th and ^40^K for 48 soil samples (dry weight) collected from various locations within the Kong Khaek subdistrict are illustrated in Figure 4b. The results of the measurement indicate that specific activity concentrations of ^226^Ra, ^232^Th and ^40^K ranged from 22.7 to 110 (with an average of 47 ± 20.9), 27.6 to 186.4 (with an average of 77. 9 ± 29.7) and 195.4 to 1108.6 Bq/kg (with an average of 700.1 ± 233), respectively. The activity concentration of natural radionuclides in all soil samples was in the following order: ^40^K > ^232^Th > ^226^Ra. The average values of ^226^Ra, ^232^Th and ^40^K exceeded the worldwide average values of 35, 30 and 400 Bq/kg, respectively [19].

Radium equivalent activity (R_eq_), an index associated with internal dose due to radon and its decay products and external gamma dose (sum of the activity of ^226^Ra, ^232^Th and ^40^K) in soil samples, is shown in Figure 4d. It is observed that the calculated value of R_eq_ ranged from 87.2 to 460.4 Bq/kg, with an average value of 212.3 ± 71.8 Bq/kg. Most of the calculated R_eq_ values were found to be lower than the recommended safe limit of 370 Bq/kg [19], except for two soil sample locations (377 and 460.4 Bq/kg).

Figure 4e displays the absorbed gamma radiation dose rate in outdoor air (D_a_) at a height of 1 m above the ground surface for 48 soil samples. The range of D_a_ in the study area was found to be from 40.7 to 208.8 nGy/h, with an average value of 98 ± 32.5 nGy/h. The calculated average value of D_a_ was higher than the worldwide average D_a_ of 59 nGy/h and the national levels of Thailand (35–44 nGy/h) [19,31].

### 3.4. Natural Radionuclide Activity Concentration in Plant Foods

All plant food samples in the study area were analyzed by gamma spectrometry system, and the results of the activity concentration of natural radionuclides (^226^Ra, ^232^Th and ^40^K) are presented in Table 1. The activity concentrations of all natural radionuclides were determined across 25 types of plants in a total of 52 samples. The measurement of specific activity concentrations of ^226^Ra was observed to range from 1.2 to 5.4 Bq/kg, with an average value of 2.7 ± 0.1 Bq/kg. The highest activity concentration of ^226^Ra was recorded for Chinese cabbage. Meanwhile, the activity concentration of ^232^Th is shown to be between 1.1 to 9.3 Bq/kg, with an average value of 3.2 ± 1.6 Bq/kg, and the highest concentration was found in celery. The activity concentration of ^40^K ranged from 338.2 to 2324.2 Bq/kg with an average value of 1000.7 ± 1.9 Bq/kg. The highest value of ^40^K concentration was found in cilantro. Interestingly, the activity concentration of ^40^K was consistent and the highest across all samples compared to the activity concentrations of ^226^Ra and ^232^Th. Overall, these results suggest that the uptake of natural radioactivity in plants is usually dependent on the type of plants.

### 3.5. Health Risk Assessment Due to Natural Background Radiation

Table 2 represents the radiological hazard parameters resulting from exposure to natural background radiation for residents of Kong Khaek. The estimated values of annual effective dose from inhalation of radon (H_Rn_) ranged from 0.5 to 3 mSv, with an average value of 1 mSv. Meanwhile, for inhalation of thoron (H_Tn_), it ranged from 0.01 to 0.6 mSv, with an average value of 0.1 mSv. The measured values of an ingestion dose contribution from radon in drinking water (H_ingw_) was found to be 0.2 to 235 μSv, with an average value of 23.3 μSv, while the inhalation dose contribution from radon in drinking water (H_RnW_) varied from 0.5 to 580 μSv with an average value of 57.4 μSv. The total annual effective dose contribution from radon in drinking water was found to vary from 0.7 to 814.8 μSv, with an average value of 80.7 μSv. The average annual effective dose from water-dissolved radon was below the MCL of 100 μSv, as reported by the WHO [32]. However, 9% of annual effective dose values were found to be above the MCL level. Moreover, the estimated value of average effective dose due to inhalation (H_Rn_ + H_Tn_ + H_RnW_) was slightly higher (1.16 mSv) than the worldwide average value of 1.1 mSv as reported by the UNSCEAR [19].

Furthermore, the concentrations of ^226^Ra, ^232^Th and ^40^K in soil samples in the study exceeded the worldwide average value. The external hazard index (H_ex_) ranged from 0.2 to 1.2, with an average value of 0.6 ± 0.2, with values mostly below the recommended value of 1. However, about 6% of H_ex_ exceeded this recommended value (Figure 4d). The outdoor annual effective dose (H_O_) ranged from 0.05 to 0.26 mSv, with an average value of 0.12 mSv. The average value of H_O_ was found to be higher than the worldwide average of 0.07 mSv as reported by the UNSCEAR [19,25].

The concentrations of ^226^Ra and ^232^Th in plant food samples were lower than the worldwide average, except ^40^K (Table 1). The internal hazard index (H_in_) was found to range from 0.03 to 0.5, with an average value of 0.2 ± 0.1. Meanwhile, the average value of H_in_ was less than the recommended value of 1. The annual effective dose received from the ingestion of food (H_ingf_) was estimated to be 0.01–0.4 mSv, with an average of 0.14 mSv. The average value of H_ingf_ was found to be lower than the worldwide average of 0.29 mSv as reported by the UNSCEAR [19].

Altogether, the average value of annual effective dose (H) was 1.4 mSv. The annual equivalent dose to lung (H_L_) and stomach (H_S_) were 3.5 and 0.4 mSv, respectively. The H_L_ value was found to be higher than the recommended limit, and the worldwide average of radiation dose from all sources of radiation. In addition, the excess lifetime cancer risk (ELCR) value was estimated to be 5.4, which is higher than the UNSCEAR recommended value of 0.29 × 10^−3^ [19]. The number of lung cancer per year per million (LCC) value due to exposure to natural background radiation was calculated to be 25.2, which is lower than the limit range of 170–230 reported by the International Commission on Radiological Protection (ICRP) publication 65 [33].

## 4. Discussion

Natural background radiation is the main sources of ionizing radiation exposure to humans and is a significant contributor to chronic health impacts caused by low dose-rate exposure, especially in the high natural background radiation area [5,6,7,8,9,10]. Chiang Mai has one of the highest natural background radiation levels in the country, as well as being one of most polluted cities in the world for air quality. This situation may result in synergistic effects, which can potentially lead to the development of LC and other health diseases [2]. In this study, we measured both external and internal radiation dose to residents due to natural background radiation.

Firstly, we measured indoor radon and thoron concentrations in 99 randomly selected houses. The measured average values of indoor radon (40.8 ± 22.6 Bq/m^3^) and thoron (17.8 ± 16.3 Bq/m^3^) concentrations in the Kong Khaek subdistrict of Chiang Mai province for 6 months were found to be higher than the worldwide average of 40 Bq/m^3^ for radon and 10 Bq/m^3^ for thoron. This could be explained by the high radon potential area of granitic bed rocks and results in the high specific activity of ^226^Ra and ^232^Th in the soil samples from the study areas (Figure 4b). Surprisingly, we found more than 80% of well-ventilated wooden houses in the study area. The maximum value of radon concentration (119 Bq/m^3^) was higher than the permissible level of 100 Bq/m^3^ as recommended by the WHO but remained below the reference levels of 300 Bq/m^3^ prescribed by ICRP. In addition, indoor thoron concentration (2–104 Bq/m^3^) in this study area cannot be neglected because of the health effects of thoron [9]. Furthermore, the results show higher indoor radon and thoron concentrations during burning season compared to during non-burning season. These average values of indoor radon (56.7 ± 20 Bq/m^3^) and thoron (20.8 ± 20.4 Bq/m^3^) concentrations were higher than the worldwide average value. This may be due to the fact that there are high concentrations of ^226^ Ra, ^232^Th and ^40^K in the soil and PM (Figure 4b,c,e) and climate parameters (such as high concentrations of indoor radon and thoron in houses during winter burning season due to a poor exchange rate in winter (Figure 2d,e). These findings align with previous reports [11,12] and emphasize the necessity of further research for indoor radon and thoron mapping during burning season and non-burning season in Chiang Mai. Table 2 presents the inhalation dose of H_Rn_ and H_Tn_ received by the residents in the investigated area. The estimated values of H_Rn_ and H_Tn_ ranged from 0.5–3 mSv (with an average value of 1 mSv) and 0.01–0.6 mSv (with an average value of 0.1 mSv), respectively. Moreover, the inhalation dose of H_Rnw_ ranged from 0.5 to 580 μSv (with an average of 57.4 μSv), and the average total inhalation dose due to radon and thoron was 1.16 mSv. The estimated dose is slightly higher than the worldwide average inhalation dose (1.1 mSv) due to radon and thoron, as reported by the UNSCEAR [19]. However, the highest total inhalation dose in this study area was more than 3 mSv and should be limited to minimized exposure to radon and thoron for the residents of these houses.

Secondly, radon concentration was measured in drinking water from underground water in Kong Khaek subdistrict. Generally, radon is found in ground water from granite in geology [14,23,32]. Prolonged consumption of water with low or high concentrations may lead to the development of LC and stomach cancer in the human body. Results show (Figure 3b) that radon concentrations in drinking water (56 samples) ranged from 0.1 to 91.9 Bq/L, with an average value of 9.1 ± 22.8 Bq/L (geometric mean value of 1.4 Bq/L). Thirteen percent of the samples exceeded the recommended limit by USEPA of 11.1 Bq/L [14]. The highest concentration of radon in drinking water samples was observed in the villages located in the mountain. The higher radon concentration in drinking water in a few study areas is mainly because of the geology consisted of granite, which may be rich in uranium and results in a high activity concentration of ^226^Ra, ^232^Th and ^40^K in the soil samples (Figure 4b). Results of the total annual effective dose contribution from radon in drinking water (ingestion and inhalation of radon) have been compared with the MCL of 100 μSv, as recommended by the WHO. Despite these findings, it has been found that the average value of the total annual effective dose contribution from radon in drinking water (80.7 μSv) was well below the permissible limit (Table 2). The highest total annual effective dose contribution from radon in drinking water in the study area was 814.8 μSv, emphasizing the important of reducing radon concentration in drinking water in the area with high radon concentration.

Thirdly, outdoor radiation was measured in soil, as soil represents an absorbed outdoor gamma dose rates to which residents of Kong Khaek subdistrict are exposed via the transfer of terrestrial radionuclides to the natural environment. The calculated average activity concentrations of terrestrial radionuclide (^226^Ra, ^232^Th and ^40^K) in soil samples (n = 48) collected from the study area exceed the world reported values (Figure 4b). The high values of activity concentrations of terrestrial radionuclide (^226^Ra and ^232^Th) may be due to the geology consisting of rock (granite) of the study area. In addition, the high value of ^40^K in soil may be attributed to the excessive use of agricultural fertilizers. Relative contributions of activity concentrations rank are in the following order: ^226^Ra (5.7%) < ^232^Th (9.4%) < ^40^K (84.9%). Furthermore, a higher concentration of ^232^Th in the soil samples leads to an increase in indoor thoron in the respective houses (Figure 2c,e) but the R_eq_ (Figure 4c) values were lower than the worldwide average. The average of D_a_ was higher than the world average value (Figure 4e). These results indicate that higher concentration of terrestrial radionuclide in collected soil samples. The fraction contributions to D_a_ were 22.2% of ^226^Ra, 48% of ^232^Th and 29.8% of ^40^K, respectively. Therefore, D_a_ was primarily dominated by ^232^Th in the location of the study area. The average value of H_ex_ was below the recommended value of 1 (Figure 4d), indicating that the majority of locations within the study area (96%) pose no radiological hazard to residents. The higher level of H_e_ (>1) may be due to the presence of granite in some locations. However, the average value of H_O_ exceeded the world average, and this indicates a potential health hazard for humans (Table 2). Collectively, these findings indicate the natural radioactivity levels in the soil samples in certain locations can have long-term implications for radiological hazards on human health from a radiobiological perspective.

Fourthly, the measurement of natural radionuclides from soil through the uptake of plant roots was studied to assess the natural radioactivity (^226^Ra, ^232^Th and ^40^K) in plants from the study area and to evaluate the radiological hazard to human health. Based on Table 1, the activity concentrations of ^226^Ra and ^232^Th in plants were lower compared to their relevant soil. Conversely, the activity concentration of ^40^K in plants was higher than in the associated soil (Figure 4b). This may be due to ^40^K being present as an important nutrient in plant fertilizers [20]. In general, our findings suggest that the uptake of naturally occurring radionuclides in plant foods depends on plant species, locational variation and agricultural management [20,34]. The average value of H_in_ was found to be less than the recommended value of 1 (Figure 5b), indicating that consumers of plants in the Kong Khaek subdistrict may be less exposed to significant radiological hazards. The average value of H_ingf_ was also found to be lower than the worldwide average. The dose contribution to H_ingf_ due to the individual radionuclides shows that ^226^Ra incurred the major contributor dose of 35.8% followed by ^232^Th (34.8%) and ^40^K (29.4%). Celery and Chinese cabbage were found to have the highest radiological hazard among plants in the study area due to the high concentrations of ^226^Ra, ^232^Th and ^40^K.

Finally, the average value of H for resident in the study area was 1.4 mSv, excluding external exposure due to cosmic radiation. It remains below the worldwide average of radiation dose received from all sources of natural background radiation (2.4 mSv) [19]. Based on the results, it can be concluded that exposure in this area is still within safe limits. However, higher background radiation levels (H~4.3 mSv) prompt the need for further investigation into the potential long-term health effects of natural background radiation from viewpoint of health risk. Meanwhile, the average values of H_L_ and H_s_ were 3.5 and 0.4 mSv, respectively. It can be concluded that radiological hazard to the stomach due to natural background radiation is within acceptable limits, but not for the lungs. The ELCR value associated with the natural background radiation was found to be higher than the UNSCEAR recommended value. This could be attributed to elevated levels of natural radioactivity in the studied area. Moreover, the LCC average of 25.2 per million persons per year was in the limit range 170–230 per million people, as reported by the ICRP [29,30]. Overall, our results will be used for future decision making in the prevention and protection of lung cancer associated with natural background radiation.

## 5. Conclusions

To our knowledge, this study represents the first comprehensive dose-related study of both internal and external radiation doses to residents of Kong Khaek subdistrict due to natural background radiation (except cosmic radiation). It provides valuable baseline data or a background reference level (database) of environmental radioactivity monitoring for purpose of radiation protection and evaluating human health risk in the future (such as biomarker for screening lung cancer risk in high natural background radiation area). The results of the study show that the total annual effective dose varied from 0.6 to 4.3 mSv, with an average value of 1.4 mSv. This indicates a significant long-term public health hazard due to natural background radiation and suggests a heightened radiation risk for residents. Future research is warranted to conduct epidemiological studies on the health impacts arising from prolonged exposure from natural background radiation sources.

## Figures and Tables

**Table 1:** Activity concentrations of natural radionuclides ^226^Ra, ^232^Th and ^40^K in plant food samples (dry weight) collected from various places in Kong Khaek subdistrict.

Common Name/Botanic Name (Sample Size = 52)	[^226^Ra] Bq/kg	[^232^Th] Bq/kg	[^40^K] Bq/kg
1. Banana/*Musa acuminata* (1)	LLD	LLD	338.2 ± 1.5
2. Beetroot/*Beta vulgaris* (1)	LLD	LLD	1272 ± 3.0
3. Bok choy/*Brassica rapa* (2)	1.9 ± 0.1	2.6 ± 0.2	1453.6 ± 2.4
4. Cabbage/*Brassica oleracea* var. *capitata* (4)	1.3 ± 0.1	1.7 ± 0.2	826 ± 2.2
5. Celery/*Apium graveolens* (1)	5.1 ± 0.1	9.3 ± 0.2	1194.5 ± 2.6
6. Chinese cabbage/*Brassica rapa* subsp. *pekinensis* (4)	5.4 ± 0.1	4.1 ± 0.2	2018.3 ± 3.1
7. Cilantro/*Coriandrum sativum* (3)	2.3 ± 0.1	2.8 ± 0.1	2324.1 ± 2.9
8. Cucumber/*Cucumis sativus* (2)	LLD	1.8 ± 0.2	1438.4 ± 2.5
9. Eggplant/*Solanum melongena* (4)	2.5 ± 0.1	2.4 ± 0.2	1188.6 ± 2.0
10. -/*Eupatorium fortunei* Turcz. (1)	4.2 ± 0.1	4.7 ± 0.1	597.3 ± 0.9
11. Garlic/*Allium sativum* (2)	LLD	1.4 ± 0.1	597.3 ± 1.4
12. Garlic chives/*Allium tuberosum* (1)	2.5 ± 0.1	8.7 ± 0.1	950 ± 1.6
13. Green brinjal/*Solanum melongena* L. (1)	LLD	1.2 ± 0.2	1080.7 ± 2.3
14. Green peas/*Pisum sativum* (1)	LLD	1 ± 0.1	761 ± 1.3
15. Maize/*Zea mays* (4)	LLD	LLD	122.5 ± 0.5
16. Onion flower/- (1)	LLD	LLD	875 ± 1.5
17. Orange/*Citrus sinensis* (1)	LLD	LLD	370.4 ± 1.0
18. Orange peel/- (1)	LLD	5.7 ± 0.1	345 ± 0.9
19. Papaya/*Carica papaya* (3)	1.2 ± 0.1	LLD	826 ± 2.2
20. Potato/*Solanum tuberosum* (2)	LLD	1.1 ± 0.2	678.7 ± 1.9
21. Pumpkin/*Cucurbita* (3)	1.5 ± 0.1	1.1 ± 0.1	1241 ± 2.4
22. Scallions/*Allium fistulosum* (1)	LLD	1.9 ± 0.2	1337.7 ± 2.3
23. Shallots/*Allium cepa gr.aggregatum* (4)	1.3 ± 0.1	1.7 ± 0.2	996 ± 2.3
24. Thai pepper/*Capsicum annuum ‘Bird’s Eye’* (2)	LLD	LLD	848.1 ± 1.5
25. Tomato/*Solanum lycopersicum* (2)	LLD	LLD	1363.6 ± 2.3
**Average**	**2.7 ± 0.1**	**3.2 ± 1.6**	**1000.7 ± 1.9**
**Geometric average**	**2.3**	**2.5**	**658**

LLD = Lower limit of detection (^226^Ra = 1 Bq/kg, ^232^Th = 1 Bq/kg and ^40^K = 5 Bq/kg).

**Table 2:** Health risk assessment due to exposure to natural background radiation for Kong Khaek residents.

Radiological Hazard Parameters	
1. The annual effective dose from inhalation of radon (H_Rn_)	1 (0.5–3) mSv
2. The annual effective dose from inhalation of thoron (H_Tn_)	0.1 (0.01–0.6) mSv
3. The annual effective dose from ingestion of water (H_ingw_)	23.3 (0.2–235) μSv
4. The annual effective dose from inhalation of radon degassing from water (H_Rnw_)	57.4 (0.5–580) μSv
5. The annual effective dose contribution from radon in drinking water (H_ingw_ + H_Rnw_)	80.7 (0.7–814.8) μSv
6. The external hazard index (H_ex_)	0.6 ± 0.2 (0.2–1.2)
7. The outdoor annual effective dose (H_O_)	0.12 (0.05–0.26) mSv
8. The internal hazard index (H_in_)	0.2 ± 0.1 (0.03–0.5)
9. The annual effective dose from ingestion of plant foods (H_ingf_)	0.14 (0.01–0.4) mSv
10. Total annual effective dose (H)	1.4 (0.6–4.3) mSv
11. The average value of annual equivalent dose to lung (H_L_)	3.5 mSv
12. The average value of annual equivalent dose to stomach (H_s_)	0.4 mSv
13. Excess lifetime cancer risk (ELCR)	5.4
14. The number of lung cancer per year per million (LCC)	25.2 × 10^−6^

## Data Availability

The data presented in this study are available from the authors on reasonable request.
